# Aryl hydrocarbon receptor signals attenuate lung fibrosis in the bleomycin-induced mouse model for pulmonary fibrosis through increase of regulatory T cells

**DOI:** 10.1186/s13075-020-2112-7

**Published:** 2020-02-07

**Authors:** Hiroshi Takei, Hidekata Yasuoka, Keiko Yoshimoto, Tsutomu Takeuchi

**Affiliations:** 1 Division of Rheumatology, Department of Internal Medicine, Keio University School of Medicine, 35 Shinanomachi, Shinjuku-ku, Tokyo, Japan; 2 Division of Rheumatology, Department of Internal Medicine, Fujita Health University School of Medicine, 1-98 Dengakugakubo, Kutsukake-cho, Aichi, Japan; 3 Clinical and Translational Research Center, Keio University Hospital, 35 Shinanomachi, Shinjuku-ku, Tokyo, Japan

**Keywords:** Interstitial lung disease, Aryl hydrocarbon receptor, Bleomycin (BLM)-induced lung fibrosis mouse model, Regulatory T cells

## Abstract

**Background:**

Interstitial lung disease (ILD) is a serious complication of connective tissue diseases (CTDs). Although immune dysregulation triggered by genetic and environmental factors is thought to provoke inflammation and subsequent fibrosis, precise mechanisms of these processes remain unclear. Recent reports suggest that activation of aryl hydrocarbon receptor (AhR) signals by various ligands such as tryptophan derivatives can induce hyper-immune responses and are involved in autoimmunity. We investigated the effects of AhR signals on the process of lung fibrosis and changes in immunological features using a bleomycin (BLM)-induced lung fibrosis mouse model.

**Methods:**

BLM was administered intratracheally to C57BL/6JJcl mice and either 5,11-dihydroindolo[3,2-b]carbazole-6-carboxaldehyde (FICZ), a natural AhR ligand, or vehicle was subsequently injected intraperitoneally on day 0, 1, and 2 from BLM administration. Mice were sacrificed at week 3, and lung fibrosis was quantified by the histological changes using the Ashcroft score and deposition of soluble collagen levels in the lung using Sircol assay. The population of immune cells infiltrated into the lungs was analyzed using flow cytometry.

**Results:**

Both the Ashcroft score and soluble collagen level in FICZ-treated mice were significantly lower than those in the vehicle group. Moreover, the survival rate of FICZ-treated mice was significantly higher than that of control mice during the 3 weeks after treatment. Interestingly, flow cytometric analysis revealed that the number of CD4^+^Foxp3^+^ regulatory T cells (Tregs) was significantly increased and CD4^+^IFNγ^+^ and γδ^+^IL-17A^+^ T cells were decreased in the lungs of FICZ-treated mice, while the total number of T, B, and NK cells were unaffected by FICZ treatment.

**Conclusions:**

Our findings suggest that stimulation of AhR signals attenuated lung fibrosis by increasing Tregs and suppressing inflammatory T cell subsets in a BLM-induced fibrosis model. AhR signaling pathways may therefore be useful therapeutic targets for connective tissue disease-associated *ILD*.

## Introduction

Interstitial lung disease (ILD) is a frequent and serious complication in patients with connective tissue diseases (CTDs) and is associated with significant morbidity and mortality [1, 2]. In addition to genetic factors, environmental factors are also thought to be important for triggering the disease progression of CTDs [3, 4]. For example, occupational exposure to silica, asbestos, organic solvents such as trichloroethylene, and aromatic solvents are risk factors for systemic sclerosis, a prototype CTD with systemic fibrosis, including ILD [5, 6]. Further, cigarette smoking is strongly associated with the disease progression of rheumatoid arthritis with ILD [7]. However, the mechanisms by which these environmental factors cause immune dysregulation and ILD have not been well elucidated. The aryl hydrocarbon receptor (AhR) is a unique molecule with an emerging role that appears to directly connect environmental factors with the immune system [8, 9]. AhR belongs to the basic helix-loop-helix PAS (PER-ARNT-SIM) domain family of transcription factors and was originally identified as a receptor targeted by xenobiotic toxins. However, subsequent studies have revealed the evolutionary conservation of the AhR and various endogenous AhR ligands such as tryptophan derivatives and indoles, suggesting physiological functions other than a xenobiotic receptor [8, 10]. Of note, recent reports have revealed that the AhR is widely expressed in the immune system and that activation of AhR by its ligands has modulatory effects on the immune system, including balancing the differentiation of regulatory T cells (Tregs) and IL-17-producing helper T cells (Th17 cells), which may be associated with autoimmunity [8, 9, 11, 12]. Furthermore, administration of AhR ligands has ameliorated the disease process of various animal models, such as experimental autoimmune encephalomyelitis and experimental colitis [11, 13–15]. Thus, we hypothesized that AhR signaling may also be involved in the process of lung fibrosis via its modulatory effects on the immune system.

## Materials and methods

### Administration of FICZ to a BLM-induced pulmonary fibrosis mouse model

Female C57BL/6JJcl (8–10 weeks) mice were purchased from Clea Japan, Inc. (Tokyo, Japan). All experiments were performed in accordance with the guidelines for animal care and use approved by Keio University School of Medicine. For BLM-induced pulmonary fibrosis, mice were intratracheally administered BLM (Nippon Kayaku, Tokyo, Japan) dissolved in a total 50 μL of phosphate-buffered saline (PBS) at a dose of 0.06 U on day 0 [16]. Control mice were administered 50 μL of PBS in the same manner. Either 1 μg of 5,11-dihydroindolo[3,2-b]carbazole-6-carboxaldehyde (FICZ) (Abcam, Cambridge, MA) dissolved in 200 μL corn oil (Sigma-Aldrich, St. Louis, MO) or vehicle was i.p. injected on day 0, 1, and 2 from BLM administration.

### Histologic analysis and Sircol assay

Mice were sacrificed at week 3 after BLM administration for the evaluation of lung fibrosis. The left lung was fixed by infiltration with 4% paraformaldehyde for 48 h and embedded in paraffin. The lung sections were prepared at 5-μm thickness from paraffin-embedded blocks and stained with hematoxylin and eosin (H&E). Lung fibrosis was histopathologically quantified using the Ashcroft score as described previously [17] at five points of each slide and the averaged score was used for the further analyses.

The deposition of soluble collagen in the right lung was analyzed using the Sircol assay according to the manufacturer’s protocol (Biocolor Ltd., Carrickfergus, UK). Briefly, the right lung was harvested and homogenized in PBS and incubated with acid-pepsin extraction media at 4 °C for 24 h. Lung extracts were incubated with Sircol dye, which binds to soluble collagen, and then centrifuged to form pellets. Pellets were solubilized in sodium hydroxide and the amount of eluted dye was measured using a microplate reader at 540 nm. Collagen standards supplied with the kit were used as controls.

### Cell isolation for flow cytometry

Mice were sacrificed at week 1 after BLM administration for the analysis of infiltrating cells in the spleen and lungs. The spleen was minced with scissors and passed through a 40-μm cell strainer. Erythrocytes were lysed with Ammonium-Chloride-Potassium Lysing Buffer and spleen cells were re-suspended in a magnetic cell sorting buffer (PBS, 0.5% bovine serum albumin and 2 mM EDTA) after washing twice with PBS. Whole lungs were excised after transcardial perfusion with PBS. Excised lungs were digested with collagenase D (1 mg/ mL; Sigma-Aldrich) and DNase (1 mg/mL; Worthington, Columbus, OH) at 37 °C for 40 min. After filtering through a cell strainer, the extract was washed with a magnetic cell sorting buffer. Erythrocytes were lysed in the same manner as the spleen and lung cells including immune cells infiltrated in the tissue were re-suspended in magnetic cell sorting buffer.

### Flow cytometry

For intracellular cytokine staining, cells were stimulated for 5 h in RPMI 1640 medium containing 10% fetal bovine serum and penicillin-streptomycin (Thermo Fisher Scientific, Waltham, MA) with PMA (1 μg/mL), ionomycin (1 μg/ mL), and Golgiplug (BD Biosciences, Franklin Lakes, NJ). Surface staining was performed for 20 min after incubating with FcBlock (BD Biosciences) for 5 min at 4 °C, and intracellular staining was subsequently performed using a Foxp3/Transcription Factor Staining Buffer Set (Thermo Fisher Scientific) according to the manufacturer’s instructions. A single-cell suspension was stained with anti-mouse CD45 (BV510, BD Biosciences), CD3ε (PE-cy5, Thermo Fisher Scientific), CD4 (eFluor® 450, Thermo Fisher Scientific), γδTCR (FITC, Thermo Fisher Scientific), CD8a (PE-Cy™, BD Biosciences), B220 (PE, Thermo Fisher Scientific), and NK-1.1 (APC, BD Biosciences) for surface staining. For intracellular staining, a single-cell suspension was stained with anti-mouse INF-γ (PE-Cyanine7, Thermo Fisher Scientific), IL-17A (PE, Thermo Fisher Scientific), IL-22 (APC, Thermo Fisher Scientific), and Foxp3 (APC, Thermo Fisher Scientific). Cells were analyzed using MACS Quant Analyzer with Flowlogic software (Miltenyi Biotec, Gladbach, Germany).

### Statistical analysis

Data were analyzed using Prism software version 6.05 (GraphPad Software, San Diego, CA). Mann-Whitney and Kaplan-Meier survival analyses were used to compare the data. Data were considered statistically significant at *p* < 0.05.

## Results

### FICZ attenuated lung fibrosis histologically and quantitatively in BLM-treated mice

To elucidate whether AhR signals affect the process of lung fibrosis induced by BLM, we used FICZ as an AhR ligand due to its lack of toxicity and high affinity for AhR [8, 10].

**Fig. 1:**
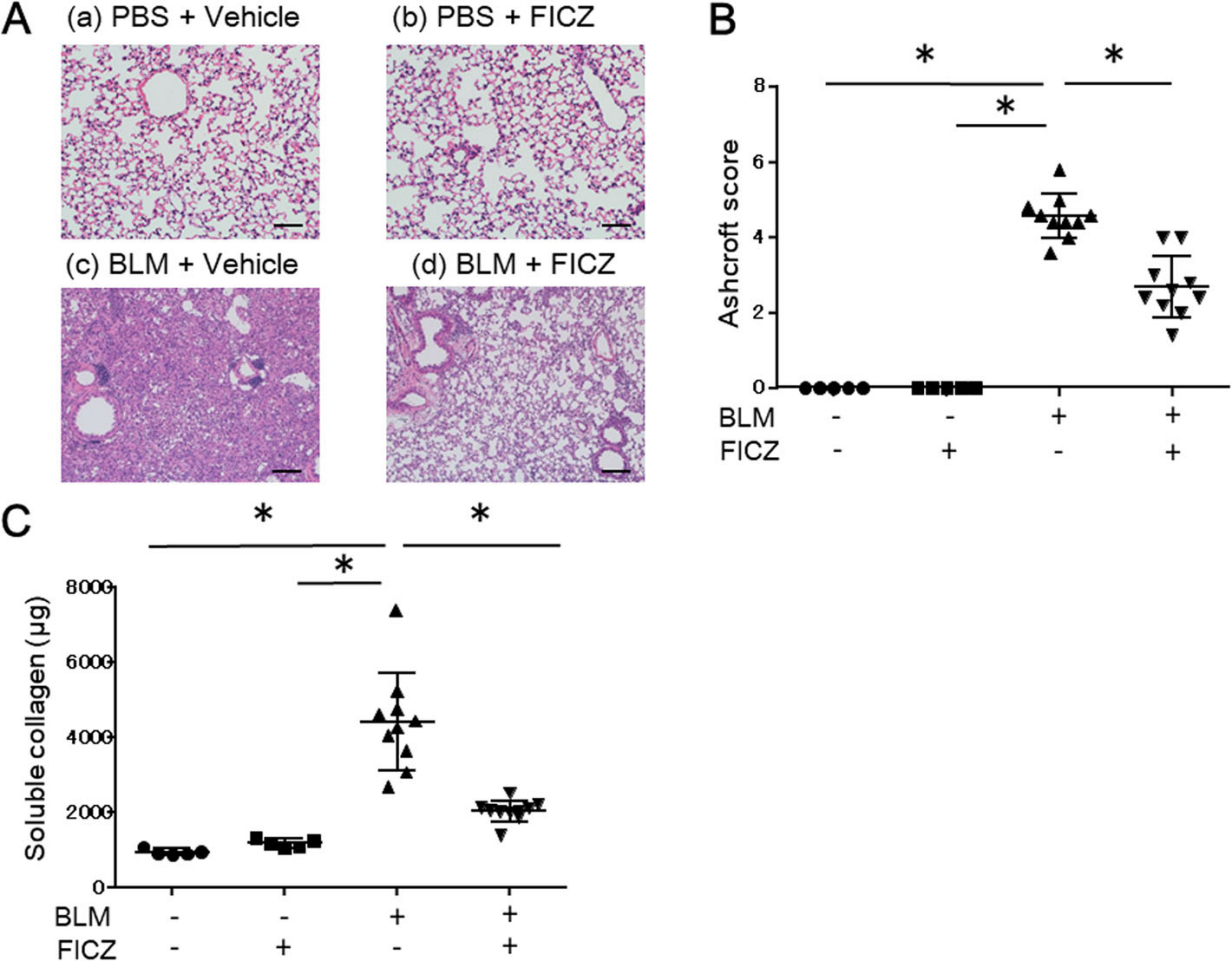
FICZ attenuated lung fibrosis in a bleomycin-induced mouse model. BLM at a dose of 0.06 units/animal was intratracheally administered with (denoted as FICZ) or without FICZ (vehicle). PBS was intratracheally administered as a control of BLM. Mice were sacrificed 3 weeks after BLM administration to evaluate lung fibrosis. Representative images of hematoxylin and eosin-stained sections of left lungs are shown for the four groups (**A** (a–d)). Magnification of the original images was × 100. And the bar denotes 100 μm. Summary of the histopathological score of left lungs (**B**) and collagen content in the right lung (**C**) in 4 groups of mice with different treatment arms was shown. *n* = 10 for BLM with or without FICZ groups and *n* = 5 for the control groups. *p < 0.05 using Mann-Whitney test

Given that BLM has been shown to induce fibrosis 2–4 weeks after intratracheal administration [18], we histologically analyzed sections of left lung tissue and the deposition of soluble collagen in right lung tissue from each group at week 3 after BLM administration. Figure 1A shows representative H&E-stained sections of left lungs. Intratracheal administration of BLM with an i.p. injection of vehicle induced marked lung fibrosis (c), which was attenuated when BLM was administered with an i.p. injection of FICZ (d). Intratracheal administration of PBS did not induce fibrosis irrespective of whether it was combined with an i.p. injection of vehicle (a) or FICZ (b). We also compared the Ashcroft score for semi-quantification of lung histopathology among the four groups (Fig. 1B). The score was significantly higher in mice treated with BLM with vehicle than those treated with PBS with vehicle or FICZ (4.6 ± 0.6 vs 0.0 ± 0.0 vs 0.0 ± 0.0, *p* < 0.01 and *p* < 0.01, respectively, *n* = 10 for BLM with vehicle and *n* = 5 for control groups). The score of FICZ-treated mice was lower compared with that of vehicle-treated mice in BLM-treated groups (2.7 ± 0.8 vs 4.6 ± 0.6, *p* < 0.01, *n* = 10 in each group).

We also measured soluble collagen deposition in the right lung using the Sircol assay (Fig. 1C). Soluble collagen levels were significantly higher in mice treated with BLM with vehicle than in those treated with PBS and BLM with FICZ (4423 ± 1296 μg vs 946 ± 82, *p* < 0.01 and 4423 ± 1296 μg vs 2042 ± 277 μg, *p* < 0.01, respectively, *n* = 10 for BLM with or without FICZ groups and *n* = 5 for control groups), suggesting that FICZ attenuated soluble collagen deposition in the process of BLM-induced lung fibrosis compared to vehicle.

### FICZ improved the survival rate of BLM-treated mice

**Fig. 2:**
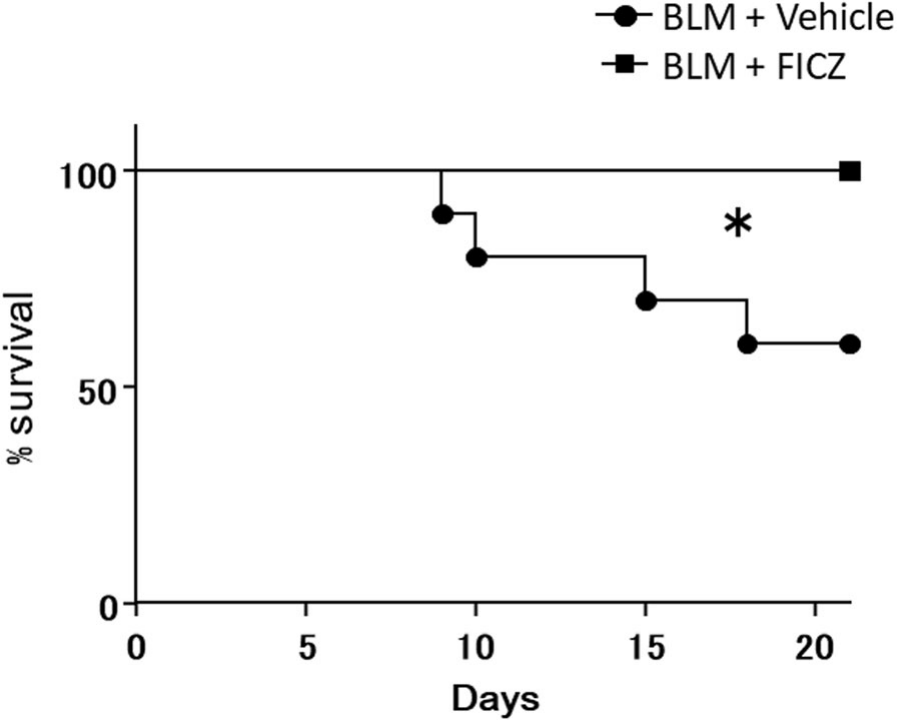
FICZ improved survival in a bleomycin-induced mouse model. BLM at 0.06 units/animal was intratracheally administered with (denoted as FICZ) or without FICZ (vehicle). Survival curves estimated by Kaplan-Meier method for the two groups are shown. *n* = 10 in each group. *p < 0.05 using the log-rank test

Furthermore, we also examined the survival of the BLM-administered mice treated with FICZ or vehicle. The survival rate of the FICZ group was significantly higher than that of the vehicle group during the 3 weeks of treatment (*p* = 0.03, *n* = 10 in each group) (Fig. 2). Taken together, these findings suggest that FICZ attenuated BLM-induced lung fibrosis and improved the survival rate of BLM-treated mice.

### FICZ increased the number of CD4^+^Foxp3^+^ Tregs among lymphocytes in the lungs of BLM-treated mice

To elucidate the mechanism underlying the attenuation of lung fibrosis in BLM-treated mice by FICZ, we analyzed the populations of immune cells that had infiltrated the lungs and spleen using FACS. C57BL/6JJcl mice (female, 8–10 weeks) were treated with BLM with FICZ or vehicle and single cells including inflammatory cells were harvested by homogenization of whole lungs and spleen at the first week after treatment, on the basis that inflammatory cells have been shown to appear within the first week [18]. The gating strategy used for spleen cells is shown in Additional file 1: Figure S1 and the defiition of cell subsets is shown in Additional file 7: Table S1; the same strategy was used for the analysis of lung cells.

**Fig. 3:**
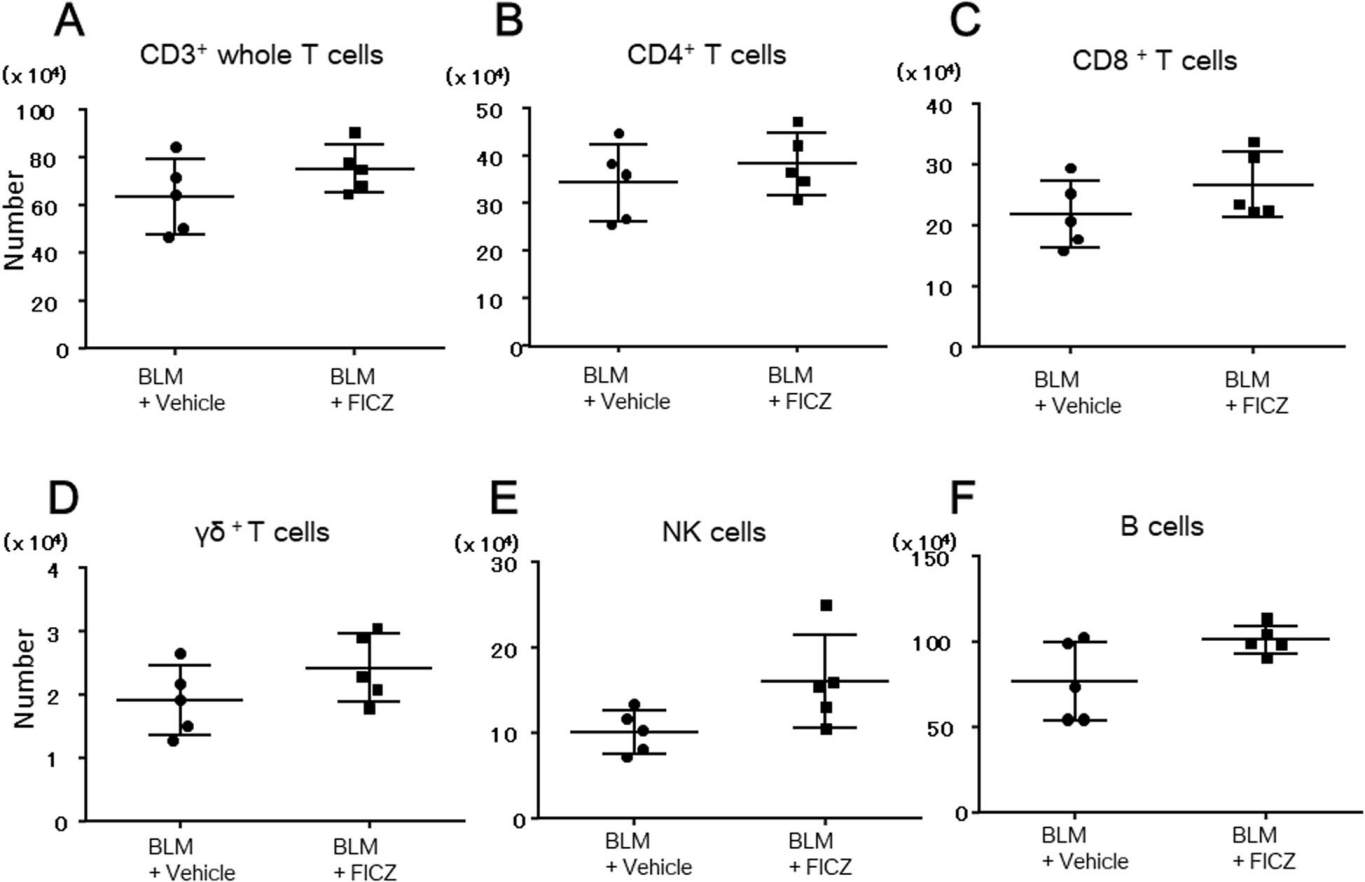
FICZ did not affect total number of several lymphocyte subsets in the lung. BLM at 0.06 units/animal was intratracheally administered with (denoted as FICZ) or without FICZ (vehicle). Flow cytometry was used to examine single-cell suspensions extracted from lung tissues stained with anti-mouse CD45, CD3, CD4, γδ, CD8, B220, NK-1.1, and viability dye 1 week after BLM administration. Total number of CD3^+^ T cells (**a**), CD3^+^CD4^+^ T cells (**b**), CD3^+^CD8^+^ T cells (**c**), CD3^+^γδ^+^ T cells (**d**), CD3^−^B220^−^NK1.1^+^ NK cells (**e**), and B220^+^ B cells (**f**) was compared between the two groups. *n* = 5 in each group using Mann-Whitney test

First, we investigated the total number of T cells, B cells, and NK cells using cell surface staining. The number of CD3^+^ T cells, CD3^+^γδ^+^ T cells, CD3^+^CD4^+^ T cells, CD3^+^CD8^+^ T cells, B220^+^ B cells and CD3^−^B220^−^NK1.1^+^ NK cells in the lungs was comparable between the FICZ and vehicle group at 1 week after BLM administration (Fig. 3a–f, *n* = 5 in each group). In the spleen also, the number of these same cell populations was comparable between the FICZ and vehicle group (Additional file 2: Figure S2A–F, *n* = 5 in each group).

**Fig. 4:**
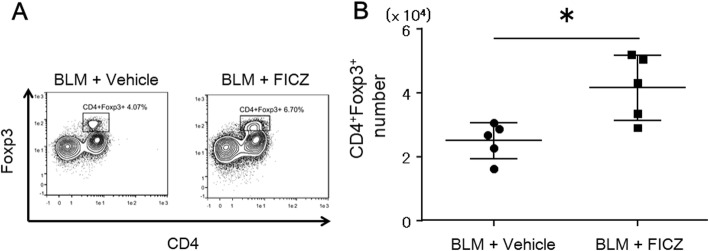
FICZ increased CD4^+^Foxp3^+^ Tregs in the lungs 1 week after BLM administration. BLM at 0.06 units/animal was intratracheally administered with (denoted as FICZ) or without FICZ (vehicle). **a** Representative flow cytometry plots of single-cell suspensions extracted from lung tissues stained with anti-mouse CD45, CD3, CD4, Foxp3, and viability dye 1 week after BLM administration are shown. **b** Summary of total number of CD4^+^Foxp3^+^ Tregs compared between the two groups. *n* = 5 in each group. *p < 0.05 using Mann-Whitney test

We also investigated the proportion of CD4^+^Foxp3^+^Tregs that had infiltrated the lungs and spleen. Interestingly, we found that FICZ had increased infiltration of CD4^+^Foxp3^+^ Tregs in the lungs (4.1 × 10^4^ ± 1.0 vs 2.5 × 10^4^ ± 0.6, *p* < 0.05, *n* = 5 in each group) at 1 week after BLM administration (Fig. 4), while the number of CD4^+^Foxp3^+^ Tregs in the spleen did not significantly differ between the FICZ and vehicle group (Additional file 3: Figure S3, *n* = 5 in each group).

### FICZ reduced the proportion of inflammatory cell subsets in the lungs

**Fig. 5:**
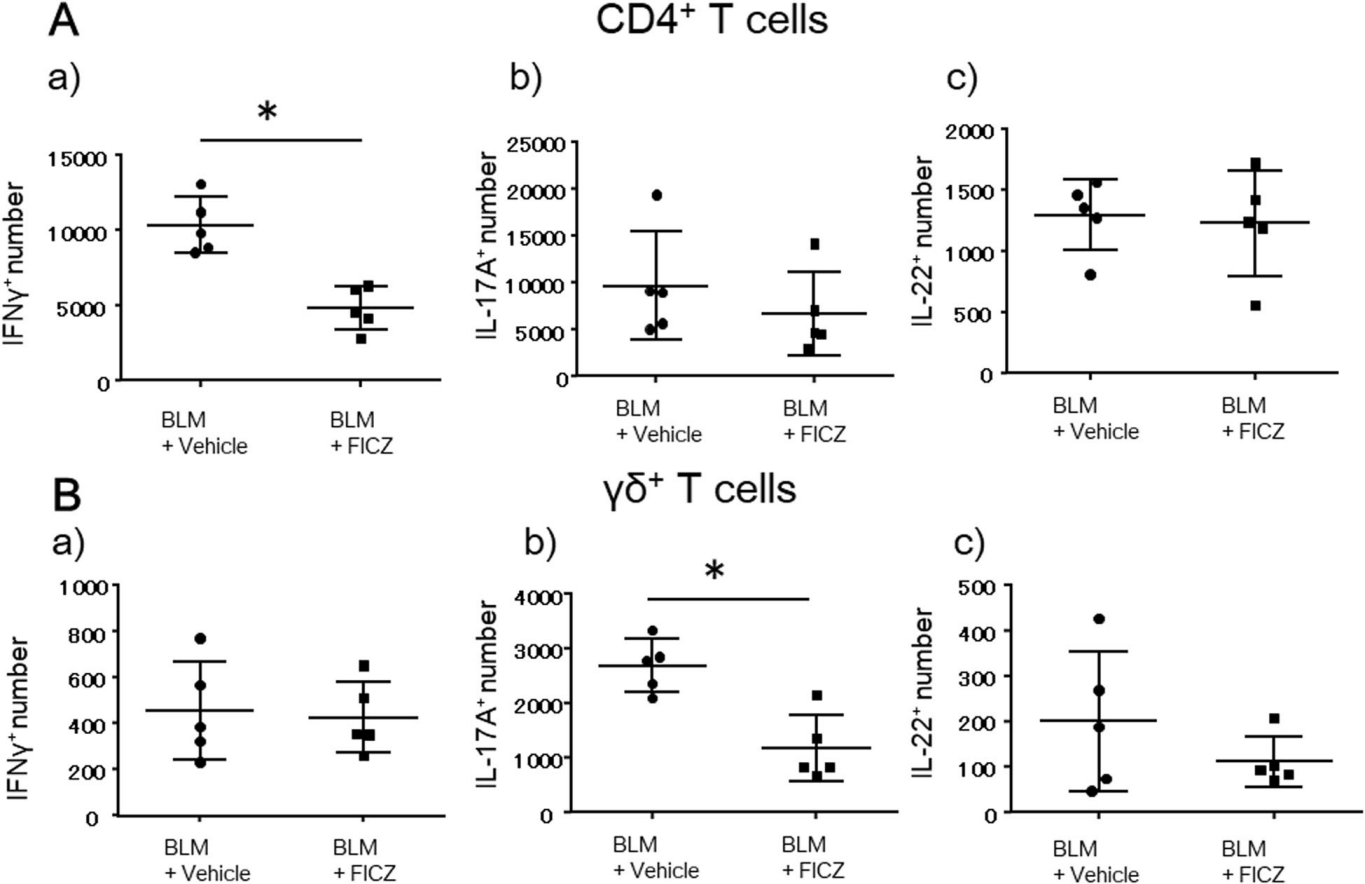
FICZ reduced CD4^+^IFNγ^+^ and γδ^+^IL-17A^+^ T cells in the lungs at 1 week. BLM at 0.06 units/animal was intratracheally administered with (denoted as FICZ) or without FICZ (vehicle). Single-cell suspensions extracted from lung tissues were stained with anti-mouse CD45, CD3, CD4, γδ, IFN-γ, IL-17A, IL-22, and viability dye 1 week after BLM administration and analyzed by flow cytometry. Summary of the number of CD4^+^ (**A**) and γδ^+^ T cells (**B**) producing the cytokines IFN-γ (**a**), IL-17A (**b**), and IL-22 (**c**) was compared between the two groups. *n* = 5 in each group. *p < 0.05 using Mann-Whitney test

We further analyzed other T cell subsets known to be involved in inflammatory responses, such as CD4^+^IFNγ^+^ T cells, CD4^+^IL-17A^+^ T cells and IL-17A^+^γδ^+^ T cells, in the lungs and spleen using flow cytometry. Intracellular staining of IFNγ, IL-17A, and IL-22 in CD4^+^ and γδ^+^ T cells revealed that FICZ reduced infiltration of CD4^+^IFNγ^+^ T cells and γδ^+^IL-17A^+^ T cells into the lungs compared to the vehicle group (4.8 × 10^3^ ± 1.4 vs 10.3 × 10^3^ ± 1.9 and 1.2 × 10^3^ ± 0.6 vs 2.7 × 10^3^ ± 0.5 respectively, *p* < 0.05; Fig. 5 and Additional file 4: Figure S4, *n* = 5 in each group). In addition, FICZ also reduced the number of CD4^+^IFNγ^+^ T cells in the spleen of FICZ-treated mice (1.5 × 10^5^ ± 0.4 vs 2.7 × 10^5^ ± 0.2, *p* < 0.05; Additional file 5: Figure S5 and Additional file 6: Figure S6, *n* = 5 in each group).

### FICZ enhanced accumulation of CD4^+^Foxp3^+^ Tregs in the lungs of BLM-treated mice during the inflammatory phase and reduced inflammatory cell subsets

We further analyzed the dynamics of CD4^+^Foxp3^+^ Tregs, CD4^+^IFNγ^+^ T cells, and γδ^+^IL-17A^+^ T cells in lung tissues and compared among BLM-treated mice with or without FICZ and PBS-treated mice with FICZ. C57BL/6JJcl mice (female, 8–10 weeks) were treated with BLM or PBS with FICZ or corn oil and the single cells from lung tissues were harvested at 1 and 3 weeks after administration.

**Fig. 6:**
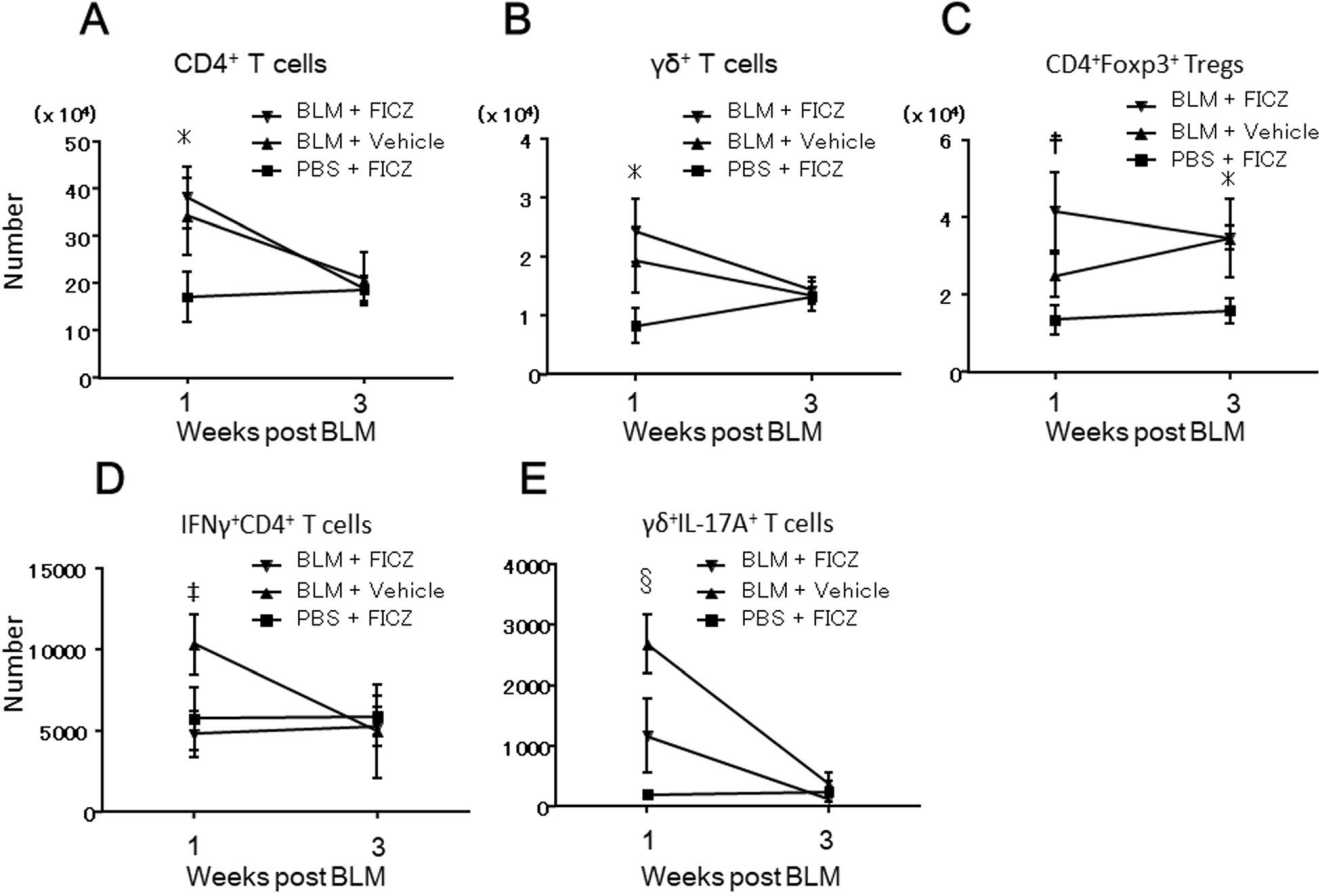
Chronologic changes in the number of Tregs, CD4^+^IFNγ^+^ and γδ^+^IL-17A^+^ T cells after BLM administration. BLM at 0.06 units/animal was intratracheally administered with (denoted as FICZ) or without FICZ (vehicle). PBS was intratracheally administered as a control of BLM. Mice were sacrificed 1 and 3 weeks after administration for flow cytometric analysis. Total number of CD3^+^CD4^+^T cells (**a**), CD3^+^γδ^+^ T cells (**b**), CD4^+^Foxp3^+^ Tregs (**c**), CD4^+^IFNγ^+^ T cells (**d**), and γδ^+^IL-17A^+^ T cells (**e**) were compared among the three groups. *n* = 5 in each group. *p < 0.05 BLM with FICZ or BLM with vehicle compared to PBS with FICZ using Mann-Whitney test. ^☨^*p* < 0.05 BLM with FICZ compared to BLM with vehicle, and BLM with vehicle compared to PBS with FICZ using Mann-Whitney test. ^‡^*p* < 0.05 BLM with vehicle compared to BLM with FICZ or PBS with vehicle using Mann-Whitney test. ^§^*p* < 0.05 BLM with vehicle compared to BLM with FICZ, and BLM with FICZ compared to PBS with FICZ using Mann-Whitney test

As shown in Fig. 6 a and b, the number of whole CD4^+^ and γδ^+^ T cells was increased in the lung of BLM-treated mice irrespective of FICZ treatment compared to those treated with PBS-treated mice at week 1 after treatment, while these were comparable among three groups at week 3 (*n* = 5 in each group).

Interestingly, the number of CD4^+^Foxp3^+^ Tregs also increased in the lungs of mice treated with BLM with vehicle compared to those treated with PBS at week 1, although the increase was greater in mice treated with BLM with FICZ. Furthermore, CD4^+^Foxp3^+^ Tregs at week 3 were comparable between BLM-treated mice with or without FICZ and higher than that of PBS-treated mice (Fig. 6c, *n* = 5 in each group). These results suggested FICZ transiently enhanced the accumulation of CD4^+^Foxp3^+^ Tregs in the lungs of BLM-treated mice especially during inflammatory phase.

Finally, the number of CD4^+^IFNγ^+^ T cells and γδ^+^IL-17A^+^ T cells increased in the lungs of mice treated with BLM with vehicle compared to those treated with PBS at week 1, and subsequently decreased to comparable levels at week 3 (*n* = 5 in each group). In contrast, FICZ reduced the number of these inflammatory subsets in the lungs of BLM-treated mice compared to the vehicle-treated group at week 1, as described above, and the number of these subsets was likewise comparable to that of PBS-treated mice at week 3 (Fig. 6d, e, *n* = 5 in each group).

Taken together, our findings suggest that administration of FICZ to BLM-induced pulmonary fibrosis model mice increased the number of CD4^+^Foxp3^+^ Tregs and reduced the number of CD4^+^IFNγ^+^ and γδ^+^IL-17A^+^ T cell populations in the lungs and contributed to improve or attenuate lung inflammation induced by BLM.

## Discussion

We demonstrated that stimulation of AhR signals by FICZ attenuated lung fibrosis and improved the survival rate of mice with pulmonary fibrosis induced by BLM. In addition, we found that FICZ increased the number of infiltrating CD4^+^Foxp3^+^Tregs in the lungs and reduced the number of inflammatory subsets, such as IFNγ^+^CD4^+^ T cells and IL-17A^+^γδ^+^ T cells, during the inflammatory phase induced by BLM. These results suggest that modulation of AhR signaling might be one of candidate pathways to regulate the process of pulmonary fibrosis or remodeling in the diseases with fibrotic phenotype such as systemic sclerosis or idiopathic pulmonary fibrosis (IPF).

There has been a discussion whether inflammation is involved in the process of fibrosis, especially in IPF, but CD4^+^ helper T cells, especially the shift to Th2, are also thought to be important for the patients with idiopathic pulmonary fibrosis [19]. While AhR can modulate a wide variety of immune system components such as T cells, dendritic cells, and innate lymphoid cells [8, 9], it was reported that AhR is important for the modulatory effects on the immune system including balancing the differentiation of regulatory T cells (Tregs) and IL-17-producing helper T cells (Th17 cells). Our study suggests that the effects of an AhR ligand, FICZ, on AhR led to increase the number of Tregs and reduced that of CD4^+^IFNγ^+^ T cells and contributed to alleviating lung fibrosis. Tregs are well known to play an important role in suppressing inflammation and excessive immune responses [20, 21]. Moreover, previous reports have revealed that Tregs also alleviate lung inflammation in mice with lung fibrosis induced by lipopolysaccharide and ovalbumin [22–24]. Given that a considerable portion of the inflammation phase also presents within the first week after the administration of BLM and leads to fibrosis in this animal model [18], facilitating the accumulation of Tregs enhanced by FICZ in the lungs may suppress this inflammatory response and alleviate fibrosis induced by BLM. Previous report revealed latent TGF-β1 treatment caused more infiltration of CD4^+^ Treg at inflamed lung lesion induced by BLM both with immunofluorescence staining and FACS analysis, suggesting infiltration of CD4^+^ Treg at inflamed lung lesion also occurred in our model [25]. We would like to conduct the immunohistochemistry or immunofluorescence in the future experiment. Several reports have also demonstrated that IFNγ is an important inflammatory mediator in the BLM-induced fibrosis. IFNγ^+^CD4^+^ T cells in the lungs and IFNγ in bronchoalveolar lavage fluid are increased following intratracheal administration of BLM, culminating in lung fibrosis. Furthermore, knocking *IFNγ* out not only alleviates inflammation but also attenuates lung fibrosis [26, 27]. Our study demonstrated decreased CD4^+^IFNγ^+^ T cells by the treatment with FICZ, both in the lungs and spleen at 1 week after BLM administration, suggesting that FICZ administration may also contribute to the alleviation of fibrosis through decrease of CD4^+^IFNγ^+^ T cells (Additional file 7). On the other hand, the increase of Tregs may directly contribute to reduce the number of CD4^+^IFNγ^+^ T cells, since previous reports have revealed that Tregs also contribute to the regulation of CD4^+^ T helper type 1 (Th1) cells, which produce IFN-γ [28, 29].

According to the previous reports, Lehmann et al. revealed that 2-(1H-Indol-3-ylcarbonyl)-4-thiazolecarboxylic acid methyl ester (ITE), one of AhR ligands, inhibits myofibroblast differentiation in vitro, suggesting that AhR signals directly alleviate fibrosis via inhibiting activation of fibroblast [30]. Moreover, regarding anti-inflammatory role, Beamer et al. showed that 2,3,7,8-Tetrachlorodibenzodioxin (TCDD), which is also one of AhR ligands, attenuated the lung inflammation induced by silica via inhibiting IL-1β production in macrophages in vivo [31]. Besides, Simonian et al. revealed that IL-22 production via AhR signaling protected lung fibrosis induced by *Bacillus subtilis* [32], whereas IL-22 was not induced by FICZ in CD4^+^ and γδ^+^ T cells in our model (Fig. 5). Taken together, we hypothesize that FICZ attenuated lung fibrosis induced by BLM via AhR and at least one of the mechanisms was expanding CD4^+^ Tregs.

The role of Tregs in pulmonary fibrosis remains controversial. Kotsianidis et al. and Shimizu et al. showed that a decrease in the number of Tregs was associated with the progression of lung fibrosis in IPF [33, 34]. In contrast, Reilkoff et al. showed that the number of Sema 7a^+^ Tregs was increased in the peripheral blood of IPF patients and that Sema 7a^+^ Tregs induced lung fibrosis in TGF-β1 transgenic mice [35]. Lo Re et al. showed that Tregs promoted lung fibrosis in a silica-induced murine lung fibrosis model [36]. Conflicting results have also been observed for Tregs in BLM-induced pulmonary fibrosis models. While some reports have shown that Tregs alleviated fibrosis [37, 38], Birjandi et al. showed that Tregs exacerbated fibrosis [39]. Birjandi et al.’s study elegantly demonstrated that the increase in the number of Tregs with administration of IL-2 complex or adoptive transfer of Tregs at BLM administration exacerbated lung fibrosis. The same study showed that this was caused by changes in Tregs from a protective to damaging phenotype during the inflammatory phase. Given that FICZ enhanced the accumulation of CD4^+^Foxp3^+^ Tregs in the lungs of BLM-treated mice during the inflammatory phase only and that BLM induced the accumulation of CD4^+^Foxp3^+^ Tregs in this lung fibrosis model (Fig. 6c), increasing the number of protective Tregs during the inflammatory phase with FICZ as shown in the present study may lead to weaken the inflammation and result in the alleviation of pulmonary fibrosis. Furthermore, because AhR signaling affects the plasticity of T cells [11, 14], modulating AhR signaling with its ligands may be an effective strategy for increasing the protective Treg population. Later administration of FICZ after week 1 can also increase Tregs at week 3; however, considering inflammation is provoked within the first week and subsided thereafter in BLM-induced mouse model [18], expanding Tregs at week 3 may not be beneficial.

Our study has several limitations. First, the BLM-induced mouse model does not completely recapitulate IPF and/or connective tissue disease-associated *interstitial lung* diseases (CTD-ILD). Although it is still controversial whether inflammation plays a substantial role in the pathogenesis, especially in IPF, as reflected by a lack of efficacy of anti-inflammatory therapy on IPF prognosis, this model has been used as a model of IPF [40] and inflammation is still thought to have a potential role and also a trigger of the process. Furthermore, anti-inflammatory therapies such as glucocorticoids, cyclophosphamide, and mycophenolate are effective against CTDs with ILD [1, 2]. Thus, we believe that our finding will be a benefit for these fibrotic lung diseases but may be more suitable for CTD-ILD than IPF. Second, while we showed that FICZ increased the number of CD4^+^Foxp3^+^ Tregs and reduced inflammatory T cell subsets such as CD4^+^IFNγ^+^ T cells and γδ^+^IL-17A^+^ T cells, we could not perform the functional analysis of these cell subsets directly, especially induction of fibrosis, due to difficulties with their isolation of infiltrating cells in the lungs whose number was quite limited.

## Conclusion

This study showed that FICZ, a natural AhR ligand, alleviated lung fibrosis and improved survival in a BLM-induced mouse model. Notably, FICZ increased the number of CD4^+^Foxp3^+^ Tregs and reduced inflammatory T cell subsets such as CD4^+^IFNγ^+^ T cells and γδ^+^IL-17A^+^ T cells in the lungs of BLM-treated mice. Allowing for several limitations, our study uniquely sheds light on the association between AhR and fibrosis with a focus on the immune system, especially T cell immunity. Taken together, our results indicate that the immunological changes induced by AhR stimulation may contribute to attenuating lung fibrosis induced by BLM.

## Supplementary information

**Additional file 1: Figure S1.** Gating strategy for flow cytometric analysis. Leukocytes were identified as positive staining of CD45 and live cells were then identified as negative staining of Viability dye. Positive staining for either B220 or CD3 was then used to identify B220^+^ B cells or CD3^+^ T cells. CD3^+^ T cells were further subdivided into CD3^+^CD4^+^ T cells, CD3^+^CD8^+^ T cells and CD3^+^γδ^+^ T cells. NK cells were identified as CD3^-^B220^-^NK1.1^+^ cells **(A)**. CD4^+^Foxp3^+^ Tregs were identified as positive staining of Foxp3 in CD3^+^CD4^+^ T cells **(B)**. CD3^+^ T cells were subdivided into either CD3^+^CD4^+^ T cells or CD3^+^γδ^+^ T cells. Production of IFN-γ, IL-17A and IL-22 in each subset was analyzed using intracellular cytokine staining for these cytokines **(C)**.

**Additional file 2: Figure S2.** FICZ did not affect total number of several lymphocyte subsets in the spleen. BLM at 0.06 units/animal was intratracheally administered with (denoted as FICZ) or without FICZ (vehicle). Flow cytometry was used to examine single cell suspensions extracted from spleen tissues stained with anti-mouse CD45, CD3, CD4, γδ, CD8, B220, NK-1.1 and viability dye 1 week after BLM administration. Total number of CD3^+^ T cells **(A)**, CD3^+^CD4^+^ T cells **(B)**, CD3^+^CD8^+^ T cells **(C)**, CD3^+^γδ^+^ T cells **(D)**, CD3^-^B220^-^NK1.1^+^ NK cells **(E)** and B220^+^ B cells **(F)** was compared between the two groups. *n* = 5 in each group.

**Additional file 3: Figure S3.** FICZ did not affect the number of CD4^+^Foxp3^+^ Tregs in the spleen. BLM at 0.06 units/animal was intratracheally administered with (denoted as FICZ) or without FICZ (vehicle). **(A)** Representative flow cytometry plots of single cell suspensions extracted from spleen tissues stained with anti-mouse CD45, CD3, CD4, Foxp3 and viability dye 1 week after BLM administration are shown. **(B)** Total number of CD4^+^Foxp3^+^ Tregs was compared between the two groups. *n* = 5 in each group.

**Additional file 4: Figure S4.** Representative plots of cytokine producing lymphocytes in lung cells at 1 week. BLM at 0.06 units/animal was intratracheally administered with (denoted as FICZ) or without FICZ (vehicle). Representative flow cytometry plots of single cell suspensions extracted from lung tissue stained with anti-mouse CD45, CD3, CD4, γδ, IFN-γ, IL-17A, IL-22 and viability dye 1 week after BLM administration are shown.

**Additional file 5: Figure S5.** Representative plots of cytokine producing lymphocytes in spleen cells at 1 week. BLM at 0.06 units/animal was intratracheally administered with (denoted as FICZ) or without FICZ (vehicle). Representative flow cytometry plots of single cell suspensions extracted from spleen tissues stained with anti-mouse CD45, CD3, CD4, γδ, IFN-γ, IL-17A, IL-22 and viability dye 1 week after BLM administration are shown.

**Additional file 6: Figure S6.** FICZ reduced CD4^+^IFNγ^+^ T cells in the spleen 1 week after BLM administration. BLM at 0.06 units/animal was intratracheally administered with (denoted as FICZ) or without FICZ (vehicle). Single cell suspensions extracted from spleen tissues were stained with anti-mouse CD45, CD3, CD4, γδ, IFN-γ, IL-17A, IL-22 and viability dye 1 week after BLM administration and analyzed by flow cytometry. Summary of the number of CD4^+^ **(A)** and γδ^+^ T cells **(B)** producing the cytokines IFN-γ **(a)**, IL-17A **(b)**, and IL-22 **(c)** was compared between the two groups. *n* = 5 in each group. * *p* < 0.05 using Mann-Whitney test.

**Additional file 7:** **Table S1.** Identified subsets of lung or spleen cells using cell-surface markers, transcription factor and cytokines.

## Data Availability

The datasets used and/or analyzed during the current study are available from the corresponding author on reasonable request.

## Abbreviations

AhR: Aryl hydrocarbon receptor
BLM: Bleomycin
CTD-ILD: Connective tissue disease-associated interstitial lung disease
CTDs: Connective tissue diseases
FICZ: 5,11-Dihydroindolo[3,2-b]carbazole-6-carboxaldehyde
ILD: Interstitial lung disease
IPF: Idiopathic pulmonary fibrosis
PBS: Phosphate-buffered saline
Th1: CD4^+^ T helper type 1
Th17 cells: IL-17-producing helper T cells
Tregs: Regulatory T cells

## References

[1] Wells AU, Denton CP (2014). Interstitial lung disease in connective tissue disease—mechanisms and management. Nat Rev Rheumatol.

[2] Marigliano B, Soriano A, Margiotta D, Vadacca M, Afeltra A (2013). Lung involvement in connective tissue diseases: a comprehensive review and a focus on rheumatoid arthritis. Autoimmun Rev.

[3] Miller FW, Alfredsson L, Costenbader KH, Kamen DL, Nelson LM, Norris JM (2012). Epidemiology of environmental exposures and human autoimmune diseases: findings from a National Institute of Environmental Health Sciences Expert Panel Workshop. J Autoimmun.

[4] Rosenblum MD, Remedios KA, Abbas AK (2015). Mechanisms of human autoimmunity. J Clin Invest.

[5] Marie I, Gehanno JF (2015). Environmental risk factors of systemic sclerosis. Semin Immunopathol.

[6] Marie I, Gehanno JF, Bubenheim M, Duval-Modeste AB, Joly P, Dominique S (2014). Prospective study to evaluate the association between systemic sclerosis and occupational exposure and review of the literature. Autoimmun Rev.

[7] Saag KG, Kolluri S, Koehnke RK, Georgou TA, Rachow JW, Hunninghake GW (1996). Rheumatoid arthritis lung disease. Determinants of radiographic and physiologic abnormalities. Arthritis Rheum.

[8] Stockinger B, Di Meglio P, Gialitakis M, Duarte JH (2014). The aryl hydrocarbon receptor: multitasking in the immune system. Annu Rev Immunol.

[9] Cella M, Colonna M (2015). Aryl hydrocarbon receptor: linking environment to immunity. Semin Immunol.

[10] Nguyen LP, Bradfield CA (2008). The search for endogenous activators of the aryl hydrocarbon receptor. Chem Res Toxicol.

[11] Quintana FJ, Basso AS, Iglesias AH, Korn T, Farez MF, Bettelli E (2008). Control of T(reg) and T(H)17 cell differentiation by the aryl hydrocarbon receptor. Nature..

[12] Noack M, Miossec P (2014). Th17 and regulatory T cell balance in autoimmune and inflammatory diseases. Autoimmun Rev.

[13] Quintana FJ, Murugaiyan G, Farez MF, Mitsdoerffer M, Tukpah AM, Burns EJ (2010). An endogenous aryl hydrocarbon receptor ligand acts on dendritic cells and T cells to suppress experimental autoimmune encephalomyelitis. Proc Natl Acad Sci U S A.

[14] Singh NP, Singh UP, Singh B, Price RL, Nagarkatti M, Nagarkatti PS (2011). Activation of aryl hydrocarbon receptor (AhR) leads to reciprocal epigenetic regulation of FoxP3 and IL-17 expression and amelioration of experimental colitis. PLoS One.

[15] Nugent LF, Shi G, Vistica BP, Ogbeifun O, Hinshaw SJ, Gery I (2013). ITE, a novel endogenous nontoxic aryl hydrocarbon receptor ligand, efficiently suppresses EAU and T-cell-mediated immunity. Invest Opthalmol Vis Sci.

[16] Della Latta V, Cecchettini A, Del Ry S, Morales MA (2015). Bleomycin in the setting of lung fibrosis induction: from biological mechanisms to counteractions. Pharmacol Res.

[17] Ashcroft T, Simpson JM, Timbrell V (1988). Simple method of estimating severity of pulmonary fibrosis on a numerical scale. J Clin Pathol.

[18] B Moore B, Lawson WE, Oury TD, Sisson TH, Raghavendran K, Hogaboam CM (2013). Animal models of fibrotic lung disease. Am J Respir Cell Mol Biol.

[19] Wynn TA (2004). Fibrotic disease and the T_H_1/T_H_2 paradigm. Nat Rev Immunol.

[20] Miyara M, Ito Y, Sakaguchi S (2014). TREG-cell therapies for autoimmune rheumatic diseases. Nat Rev Rheumatol.

[21] Schmidt A, Oberle N, Krammer PH (2012). Molecular mechanisms of treg-mediated T cell suppression. Front Immunol.

[22] D’Alessio FR, Tsushima K, Aggarwal NR, West EE, Willett MH, Britos MF (2009). CD4+CD25+Foxp3+ Tregs resolve experimental lung injury in mice and are present in humans with acute lung injury. J Clin Invest.

[23] Schreiber TH, Wolf D, Tsai MS, Chirinos J, Deyev VV, Gonzalez L (2010). Therapeutic Treg expansion in mice by TNFRSF25 prevents allergic lung inflammation. J Clin Invest.

[24] Garibaldi BT, D’Alessio FR, Mock JR, Files DC, Chau E, Eto Y (2013). Regulatory T cells reduce acute lung injury fibroproliferation by decreasing fibrocyte recruitment. Am J Respir Cell Mol Biol.

[25] Tang YJ, Xiao J, Huang XR, Zhang Y, Yang C, Meng XM (2014). Latent transforming growth factor-β1 protects against bleomycin-induced lung injury in mice. Am J Respir Cell Mol Biol.

[26] Chen ES, Greenlee BM, Wills-Karp M, Moller DR (2001). Attenuation of lung inflammation and fibrosis in interferon-gamma-deficient mice after intratracheal bleomycin. Am J Respir Cell Mol Biol.

[27] Segel MJ, Izbicki G, Cohen PY, Or R, Christensen TG, Wallach-Dayan SB (2003). Role of interferon-gamma in the evolution of murine bleomycin lung fibrosis. Am J Physiol Lung Cell Mol Physiol.

[28] Siegmund K, Feuerer M, Siewert C, Ghani S, Haubold U, Dankof A (2005). Migration matters: regulatory T-cell compartmentalization determines suppressive activity in vivo. Blood..

[29] Sarween N, Chodos A, Raykundalia C, Khan M, Abbas AK, Walker LS (2004). CD4+CD25+ cells controlling a pathogenic CD4 response inhibit cytokine differentiation, CXCR-3 expression, and tissue invasion. J Immunol.

[30] Lehmann GM, Xi X, Kulkarni AA, Olsen KC, Pollock SJ, Baglole CJ (2011). The aryl hydrocarbon receptor ligand ITE inhibits TGFβ1-induced human myofibroblast differentiation. Am J Pathol.

[31] Beamer CA, Seaver BP, Shepherd DM (2012). Aryl hydrocarbon receptor (AhR) regulates silica-induced inflammation but not fibrosis. Toxicol Sci.

[32] Simonian PL, Wehrmann F, Roark CL, Born WK, O’Brien RL, Fontenot AP (2010). δ T cells protect against lung fibrosis via IL-22. J Exp Med.

[33] Kotsianidis I, Nakou E, Bouchliou I, Tzouvelekis A, Spanoudakis E, Steiropoulos P (2009). Global impairment of CD4+CD25+FOXP3+ regulatory T cells in idiopathic pulmonary fibrosis. Am J Respir Crit Care Med.

[34] Shimizu Y, Dobashi K, Endou K, Ono A, Yanagitani N, Utsugi M (2010). Decreased interstitial FOXP3(+) lymphocytes in usual interstitial pneumonia with discrepancy of CXCL12/CXCR4 axis. Int J Immunopathol Pharmacol.

[35] Reilkoff RA, Peng H, Murray LA, Peng X, Russell T, Montgomery R (2013). Semaphorin 7a+ regulatory T cells are associated with progressive idiopathic pulmonary fibrosis and are implicated in transforming growth factor-β1-induced pulmonary fibrosis. Am J Respir Crit Care Med.

[36] Lo Re S, Lecocq M, Uwambayinema F, Yakoub Y, Delos M, Demoulin JB (2011). Platelet-derived growth factor-producing CD4+ Foxp3+ regulatory T lymphocytes promote lung fibrosis. Am J Respir Crit Care Med.

[37] Boveda-Ruiz D, D’Alessandro-Gabazza CN, Toda M, Takagi T, Naito M, Matsushima Y (2013). Differential role of regulatory T cells in early and late stages of pulmonary fibrosis. Immunobiology.

[38] Kamio K, Azuma A, Matsuda K, Usuki J, Inomata M, Morinaga A (2018). Resolution of bleomycin-induced murine pulmonary fibrosis via a splenic lymphocyte subpopulation. Respir Res.

[39] Birjandi SZ, Palchevskiy V, Xue YY, Nunez S, Kern R, Weigt SS (2016). CD4(+)CD25(hi)Foxp3(+) cells exacerbate bleomycin-induced pulmonary fibrosis. Am J Pathol.

[40] Richeldi L, Collard HR, Jones MG (2017). Idiopathic pulmonary fibrosis. Lancet.

