# Supplementary figures and images for: Aryl hydrocarbon receptor signals attenuate lung fibrosis in the bleomycin-induced mouse model for pulmonary fibrosis through increase of regulatory T cells
Source: Arthritis Res Ther. 2020 Feb 7;22:20. doi: 10.1186/s13075-020-2112-7 (PMC7006193; doi:10.1186/s13075-020-2112-7)

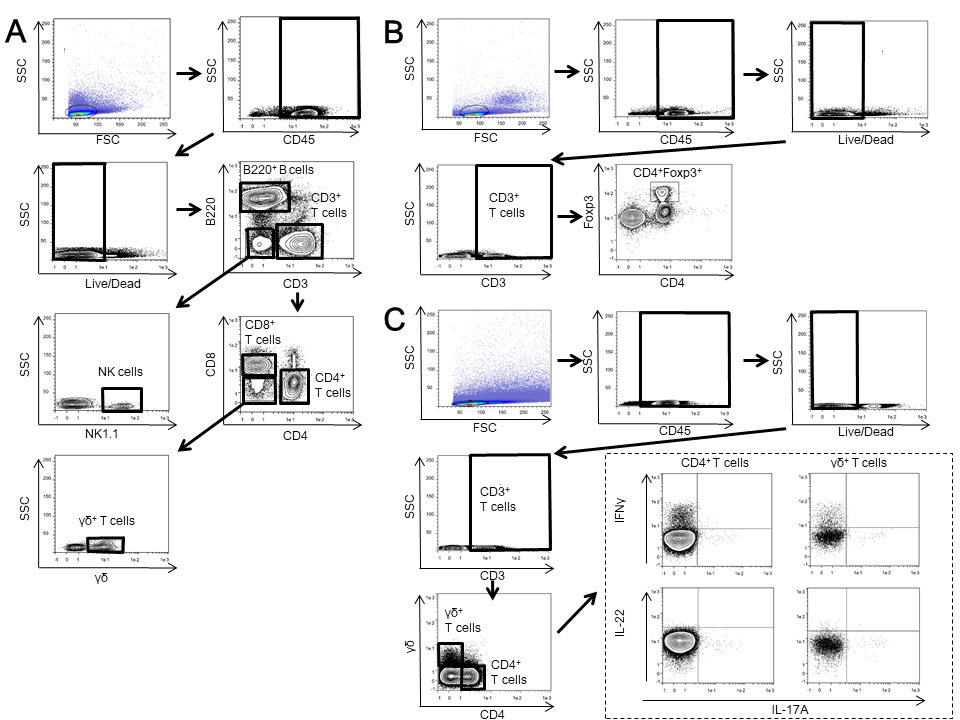

Supplement: Supplementary file 1 — Additional file 1: Figure S1. Gating strategy for flow cytometric analysis. Leukocytes were identified as positive staining of CD45 and live cells were then identified as negative staining of Viability dye. Positive staining for either B220 or CD3 was then used to identify B220+ B cells or CD3+ T cells. CD3+ T cells were further subdivided into CD3+CD4+ T cells, CD3+CD8+ T cells and CD3+γδ+ T cells. NK cells were identified as CD3-B220-NK1.1+ cells (A). CD4+Foxp3+ Tregs were identified as positive staining of Foxp3 in CD3+CD4+ T cells (B). CD3+ T cells were subdivided into either CD3+CD4+ T cells or CD3+γδ+ T cells. Production of IFN-γ, IL-17A and IL-22 in each subset was analyzed using intracellular cytokine staining for these cytokines (C). [file 13075_2020_2112_MOESM1_ESM.tif]

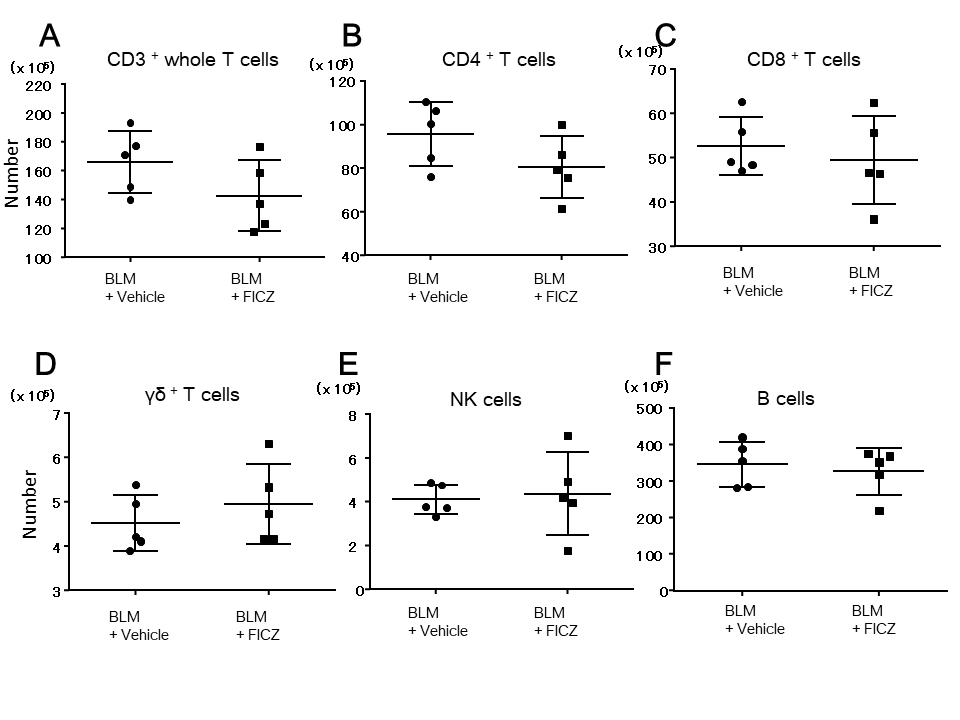

Supplement: Supplementary file 2 — Additional file 2: Figure S2. FICZ did not affect total number of several lymphocyte subsets in the spleen. BLM at 0.06 units/animal was intratracheally administered with (denoted as FICZ) or without FICZ (vehicle). Flow cytometry was used to examine single cell suspensions extracted from spleen tissues stained with anti-mouse CD45, CD3, CD4, γδ, CD8, B220, NK-1.1 and viability dye 1 week after BLM administration. Total number of CD3+ T cells (A), CD3+CD4+ T cells (B), CD3+CD8+ T cells (C), CD3+γδ+ T cells (D), CD3-B220-NK1.1+ NK cells (E) and B220+ B cells (F) was compared between the two groups. n = 5 in each group. [file 13075_2020_2112_MOESM2_ESM.tif]

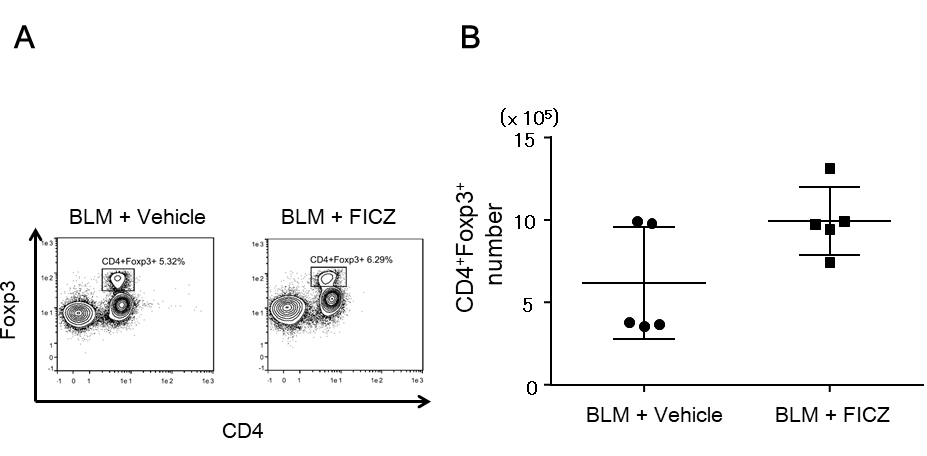

Supplement: Supplementary file 3 — Additional file 3: Figure S3. FICZ did not affect the number of CD4+Foxp3+ Tregs in the spleen. BLM at 0.06 units/animal was intratracheally administered with (denoted as FICZ) or without FICZ (vehicle). (A) Representative flow cytometry plots of single cell suspensions extracted from spleen tissues stained with anti-mouse CD45, CD3, CD4, Foxp3 and viability dye 1 week after BLM administration are shown. (B) Total number of CD4+Foxp3+ Tregs was compared between the two groups. n = 5 in each group. [file 13075_2020_2112_MOESM3_ESM.tif]

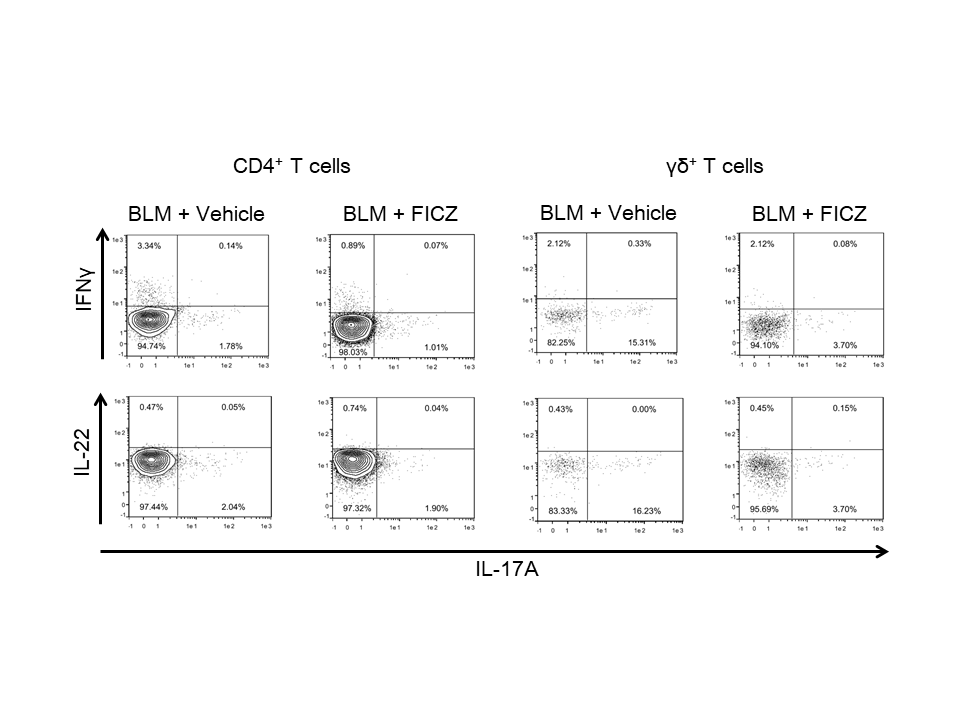

Supplement: Supplementary file 4 — Additional file 4: Figure S4. Representative plots of cytokine producing lymphocytes in lung cells at 1 week. BLM at 0.06 units/animal was intratracheally administered with (denoted as FICZ) or without FICZ (vehicle). Representative flow cytometry plots of single cell suspensions extracted from lung tissue stained with anti-mouse CD45, CD3, CD4, γδ, IFN-γ, IL-17A, IL-22 and viability dye 1 week after BLM administration are shown. [file 13075_2020_2112_MOESM4_ESM.tif]

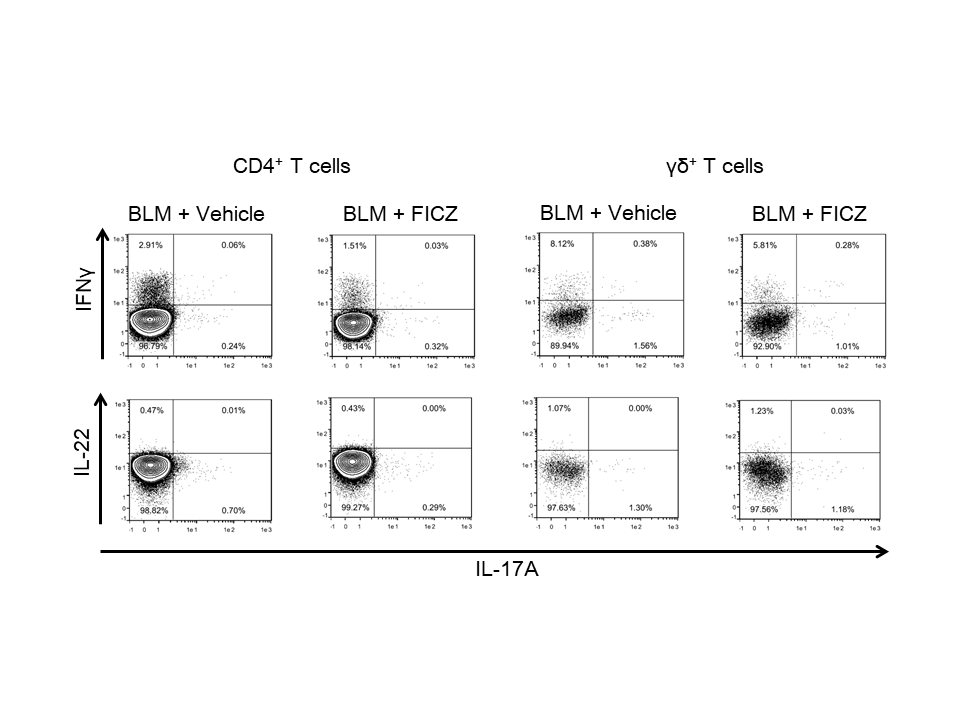

Supplement: Supplementary file 5 — Additional file 5: Figure S5. Representative plots of cytokine producing lymphocytes in spleen cells at 1 week. BLM at 0.06 units/animal was intratracheally administered with (denoted as FICZ) or without FICZ (vehicle). Representative flow cytometry plots of single cell suspensions extracted from spleen tissues stained with anti-mouse CD45, CD3, CD4, γδ, IFN-γ, IL-17A, IL-22 and viability dye 1 week after BLM administration are shown. [file 13075_2020_2112_MOESM5_ESM.tif]

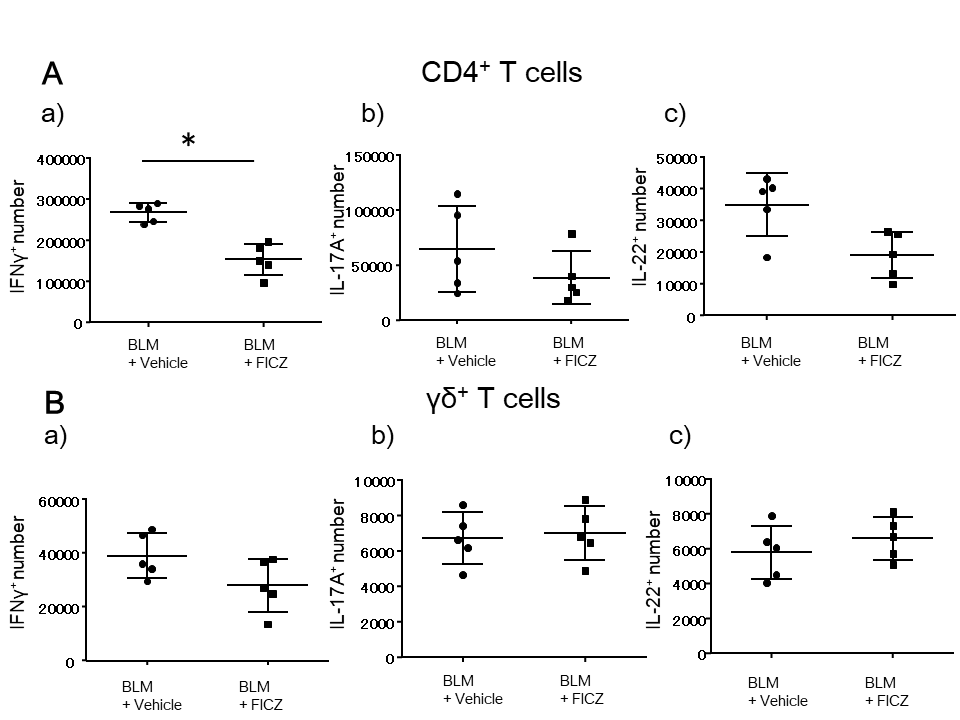

Supplement: Supplementary file 6 — Additional file 6: Figure S6. FICZ reduced CD4+IFNγ+ T cells in the spleen 1 week after BLM administration. BLM at 0.06 units/animal was intratracheally administered with (denoted as FICZ) or without FICZ (vehicle). Single cell suspensions extracted from spleen tissues were stained with anti-mouse CD45, CD3, CD4, γδ, IFN-γ, IL-17A, IL-22 and viability dye 1 week after BLM administration and analyzed by flow cytometry. Summary of the number of CD4+ (A) and γδ+ T cells (B) producing the cytokines IFN-γ (a), IL-17A (b), and IL-22 (c) was compared between the two groups. n = 5 in each group. * p < 0.05 using Mann-Whitney test. [file 13075_2020_2112_MOESM6_ESM.tif]
