# Supplementary material for: Aryl hydrocarbon receptor signals attenuate lung fibrosis in the bleomycin-induced mouse model for pulmonary fibrosis through increase of regulatory T cells
Source: Arthritis Res Ther. 2020 Feb 7;22:20. doi: 10.1186/s13075-020-2112-7 (PMC7006193; doi:10.1186/s13075-020-2112-7)
Supplement: Supplementary file 7 — Additional file 7: Table S1. Identified subsets of lung or spleen cells using cell-surface markers, transcription factor and cytokines. [file 13075_2020_2112_MOESM7_ESM.docx]

**Table S1. Identified subsets of lung or spleen cells using cell-surface markers, transcription factor and cytokines.**

| **Identified subsets** | **Definition** |
| --- | --- |
| T cells | CD45^+^CD3^+^ |
| CD4^+^ T cells | CD45^+^CD3^+^CD4^+^ |
| CD8^+^ T cells | CD45^+^CD3^+^CD8^+^ |
| γδ^+^ T cells | CD45^+^CD3^+^γδ^+^ |
| NK cells | CD45^+^CD3^-^B220^-^NK1.1^+^ |
| B cells | CD45^+^B220^+^ |
| CD4^+^Foxp3^+^ Tregs | CD45^+^CD3^+^CD4^+^Foxp3^+^ |
| CD4^+^IFNγ^+^ T cells | CD45^+^CD3^+^CD4^+^ IFNγ^+^ |
| CD4^+^IL-17A^+^ T cells | CD45^+^CD3^+^CD4^+^IL-17A^+^ |
| CD4^+^IL-22^+^ T cells | CD45^+^CD3^+^CD4^+^IL-22A^+^ |
| γδ^+^IFNγ^+^ T cells | CD45^+^CD3^+^γδ^+^IFNγ^+^ |
| γδ^+^IL-17A^+^ T cells | CD45^+^CD3^+^γδ^+^IL-17A^+^ |
| γδ^+^IL-22^+^ T cells | CD45^+^CD3^+^γδ^+^IL-22A^+^ |
